# Sri Lanka's response to prescribed drug misuse: is it enough?

**DOI:** 10.1192/bji.2021.54

**Published:** 2023-02

**Authors:** Aruni Hapangama, K. A. L. A. Kuruppuarachchi

**Affiliations:** 1 Senior Lecturer, Department of Psychiatry, Faculty of Medicine, University of Kelaniya, Sri Lanka. Email ahapangama@kln.ac.lk; 2 Senior Professor and Cadre Chair, Department of Psychiatry, Faculty of Medicine, University of Kelaniya, Sri Lanka

**Keywords:** Prescription drugs, misuse, dependence, regulations, prescribers

## Abstract

A wide range of medications are being misused by people the world over and Sri Lanka is no exception. Reasons for this misuse are manyfold. Regulatory bodies, prescribers, dispensers, as well as the general public, have significant roles to play in mitigating the misuse of prescribed medications and their harmful consequences.

Many prescription medications are being misused by people across the world, who have discovered that they can get ʻhigh' by taking them.^1^ Such misuse has been associated with significant morbidity and mortality and has been a recognised and well-documented phenomenon over the past 50 years.^1–3^ According to a United Nations World Drug Report, pharmaceutical agents that are being commonly misused are used either as adjuncts to, or replacements for, illicit narcotics and psychotropics.^4^

## The gravity of the problem in Sri Lanka

In Sri Lanka, Ayurvedic medicinal practitioners have been using cannabis and opium as therapeutic agents for centuries. However, no measures were initiated to address the non-medicinal use of psychoactive agents in Sri Lanka until the late 1970s.^5^ Even though clinicians seem to acknowledge that prescription medication misuse is a massive problem in their everyday clinical practice, there is a significant lack of published data about this issue in Sri Lanka.^4,6^ The commonly misused psychotropic agents in Sri Lanka include: pethidine, tramadol, various benzodiazepines (including diazepam, clonazepam, lorazepam, alprazolam and oxazepam), cough mixtures containing dextromethorphan, the antipsychotic quetiapine, and anticonvulsants cum chronic pain medications such as pregabalin and gabapentin.^4,6–8^

## Problems in legislation

Sri Lanka has several legislations regarding drug control and these include the Penal Code, the Poisons, Opium and Dangerous Drugs Act, the Cosmetic Devices and Drugs Act, the Customs Ordinance and the Indigenous Medicine Act.^9,10^ However, there is poor implementation of these codes and acts. As far as we are aware, there are no clear regulations in place to monitor medication prescription and to define the maximum number of medications per prescription in Sri Lanka. In countries such as the USA, to prescribe medications that are considered to have a high potential to be misused, it is mandatory that the prescriber has a licence to prescribe such medication and the pharmacist must have a licence to dispense it.^11^ Unfortunately, no such rules or regulations, although present to a certain extent, are practised widely in Sri Lanka, which also contributes to this problem. In addition, to our best knowledge, there are hardly any regulations in place to monitor the dispenser (pharmacist) in Sri Lanka in any manner and the meagre rules that are in existence are applied extremely rarely.

## Lax attitudes of prescribers and pharmacists

The apex body of medical practitioners in the country, the Sri Lanka Medical Council (SLMC), issues a registration number to all medical practitioners who are eligible to practise and prescribe in Sri Lanka. This number is expected to be inserted/written on any prescription written/issued by that practitioner. However, some prescribers are known to issue prescriptions without their prescriber number and pharmacists are known to dispense medications for prescriptions written without an SLMC registration number.

We are aware that some countries require clinicians to obtain ʻauthority' to prescribe certain medications, such as antipsychotics, and/or require the prescriber to mention the indication for prescribing a particular medication.^12,13^ However, in Sri Lanka, no such rules apply, which in our opinion results in the lax attitude of the prescribers to writing off-label prescriptions for various benzodiazepines, quetiapine, gabapentin and pregabalin. Furthermore, prescribers are rarely taken to task for such habits, which may also result in the continuation of these substandard practices.

To the best of our knowledge, a pharmacist practising in Sri Lanka should not only have registration but also apply for a certificate of good standing from the SLMC. However, not all currently practising pharmacists appear to be following these stipulated guidelines.

## Lack of trained mental health personnel

The lack of a workforce in Sri Lanka to deal with the health needs of its population has placed a large case-load per clinician and this affects mental health services more than any other discipline of medicine in this country.^14^ Under such circumstances, psychological treatment methods, especially those indicated for various anxiety, depressive and sleep disorders, which may take a longer clinician–patient time than prescribing medication, may appear non-cost-effective to some clinicians. This unfortunately results not only in the patients receiving substandard care but also in some of these patients continuing such medications for years without an authorised prescription or monitoring, leading them to develop a dependence on these agents.

## The role of the patient

In addition to the lack of regulations and the practices of prescribers and the pharmacists, another reason for prescription medication misuse in Sri Lanka is the self-medication habits of the public, who at times procure medications over the counter without a valid prescription and at times use medications prescribed for another ailment or even prescribed for someone else. On certain occasions, patients continue to take a medication for years on end without any monitoring by the prescriber.

## The impact of the COVID-19 pandemic

The ongoing COVID-19 pandemic may have had an impact on the availability and the price of illegal drugs on the streets, which may divert people who misuse or are dependent on them to use over-the-counter medications for a far cheaper price. This has been reported to have occurred in other countries^15^ and is seen in our day-to-day clinical practice in Sri Lanka.

In addition, the health regulations and travel restrictions imposed to contain the pandemic have resulted in patients not being able to be reviewed by their doctors, resulting at times in patients obtaining their medications through improper means.

## Solutions

### Stricter regulations in prescribing medications

We acknowledge that the illicit and or improper use of pharmaceutical agents is a difficult problem to address, especially in light of the lack of resources to implement and monitor them. However, we believe that proper and stricter legislation and measures such as those practised in other countries in prescribing medications should be introduced in Sri Lanka, so that people with genuine illnesses do not go without their much-needed treatments.^11,16,17^

### Regulation of pharmacies and pharmacists

To our knowledge, Sri Lanka has a large number of unlicensed pharmacies and these need to be brought under a central regulatory agency, as they are one of the main sources supplying pharmaceutical agents illegally.^5^ Audits should be conducted on pharmacists’ qualifications and the regular updating of their good standing certificate. Pharmacists should be empowered to decline to dispense medications that should not be dispensed over the counter. Stricter regulations should be put in place so that medications are dispensed only when written/issued by a registered medical practitioner who has the authority to prescribe the given medication.^5^

In Sri Lanka, the issuing of licences for pharmacies needs to be streamlined and pharmacists need to be encouraged to register themselves and operate within the said regulations.^5^

Laws must be strengthened to control dispensing and to improve the monitoring of the dispensing network in Sri Lanka. We suggest an online prescription drug monitoring system that will integrate pharmacists and medical practitioners.

In addition, pharmacists and industry must be brought under a monitoring or a regulatory authority so that they extend full cooperation to check the diversion and misuse of pharmaceutical drugs.

### Educating and empowering regulatory and law enforcement officials

Programmes should be conducted for empowering and capacity building of regulatory and law enforcement officials in Sri Lanka. Furthermore, regulatory agencies should be equipped with enough staff to implement the regulations.

### Improving public awareness

Up to now, in Sri Lanka, most public awareness campaigns have focused only on misuse and/or dependence on illicit substances. Authorities and clinicians need to be made aware that some of the ʻlegally' available drugs can be more addictive and act as a gateway to use of illegal drugs. Awareness campaigns about prescription drug misuse and its harmful consequences should be conducted regularly, given their easy availability in Sri Lanka. Similar campaigns have been reported to be effective elsewhere, especially among teenagers, who are one of the most vulnerable groups to misuse such agents.^18^

## Research

Proper and robust studies regarding the diversion of narcotics and psychotropic agents need to be conducted in all areas and settings of the country and not just in the prison system.^6^ This would provide much-needed data regarding the gravity of this problem and help in tightening the regulations and improving prescribing and dispensing practices.

We believe that prescription drug misuse has reached epidemic proportions in Sri Lanka and all stakeholders, including authorities, regulatory bodies, prescribers, dispensers and the general public, should be held responsible and accountable for mitigating this menace.

## Data Availability

Data availability is not applicable to this article as no new data were created or analysed in this study.

## References

[1] Galea S, Nandi A, Vlahov D. The social epidemiology of substance use. Epidemiol Rev 2004; 26: 36–52. 15234946 10.1093/epirev/mxh007

[2] Dunlop D. The use and abuse of psychotropic drugs. Scott Med J 1971; 16: 345–9. 5566511 10.1177/003693307101600803

[3] Bayer I. The Abuse of Psychotropic Drugs. United Nations office on Drugs and Crime, 1973.

[4] United Nations Office on Drugs and Crime. The 2009 World Drug Report. UNODC, 2009.

[5] United Nations office of Drugs and Crime. Misuse of Prescription Drugs: A South Asia Perspective. UNODC, 2011.

[6] Dissabandara LO, Dias SR, Dodd PR, Stadlin A. Patterns of substance use in male incarcerated drug users in Sri Lanka. Drug Alcohol Rev 2009; 28: 600–7. 19930012 10.1111/j.1465-3362.2009.00062.x

[7] Hapangama A, Kuruppuarachchi K. Dextromethorphan abuse. Ceylon Med J 2008; 53: 109–10. 18982808 10.4038/cmj.v53i3.256

[8] Hapangama A, Kuruppuarachchi KALA. Pregabalin misuse: a silent epidemic. Sri Lanka J Psychiatry 2019; 10(2): 15–6.

[9] Ministry of Health Care and Nutrition. The National Medicinal Drug Policy for Sri Lanka 2005. Ministry of Health Care and Nutrition, 2005.

[10] National Medicine Regulatory Authority. Guideline on good pharmacy practice (GPP). NMRA, 2019.

[11] Gabay M. Federal controlled substances act: controlled substances prescriptions. Hosp Pharm 2013; 48: 644–5. 24421533 10.1310/hpj4808-644 PMC3847977

[12] Garada M, McLachlan AJ, Schiff GD, Lehnbom EC. What do Australian consumers, pharmacists and prescribers think about documenting indications on prescriptions and dispensed medicines labels? A qualitative study. BMC Health Serv Res 2017; 17(1): 734. 29141618 10.1186/s12913-017-2704-3 PMC5688705

[13] Dresser R, Frader J. Off-label prescribing: a call for heightened professional and government oversight. J Law Med Ethics 2009; 37: 476–396. 19723258 10.1111/j.1748-720X.2009.00408.x PMC2836889

[14] Jenkins R, Mendis J, Cooray S, Cooray M. Integration of mental health into primary care in Sri Lanka. Ment Health Fam Med 2012; 9: 15–24. 23277794 PMC3487606

[15] Chiappini S, Guirguis A, Corkery GM, Schifano F. Misuse of prescription and over-the-counter drugs to obtain illicit highs: how pharmacists can prevent abuse. Pharm J 2020: 17 Nov (10.1211/PJ.2020.20208538).

[16] Clinical Guidelines on Drug Misuse and Dependence Update 2017 Independent Expert Working Group. Drug Misuse and Dependence: UK Guidelines on Clinical Management. Department of Health, 2017.

[17] Crowley DM, Jones DE, Coffman DL, Greenberg MT. Can we build an efficient response to the prescription drug abuse epidemic? Assessing the cost effectiveness of universal prevention in the PROSPER trial. Prev Med 2014; 62: 71–7. 24521531 10.1016/j.ypmed.2014.01.029 PMC4131945

[18] Spoth R, Trudeau L, Shin C, Redmond C. Long-term effects of universal preventive interventions on prescription drug misuse. Addiction 2008; 103: 1160–8. 18557842 10.1111/j.1360-0443.2008.02160.x

